# Diet and Nutrition: Eye on Iodine

**DOI:** 10.1289/ehp.116-a200a

**Published:** 2008-05

**Authors:** Cynthia Washam

Before the 1924 advent of salt iodization, millions of Americans suffered from iodine deficiency. Now, eight decades after salt iodization virtually eliminated iodine deficiency in the United States, intake of this essential mineral has once again declined enough for some scientists to recommend new measures to ensure Americans get sufficient amounts.

The thyroid gland need iodine in order to produce thyroid hormone, which regulates body metabolism, growth, and development. Iodine is especially important during pregnancy and lactation for infants’ neurological development. Maternal iodine deficiency is associated with mental retardation and cretinism in the newborn.

In the first National Health and Nutrition Examination Survey (NHANES I), conducted in the early 1970s, only 1% of pregnant women examined had urinary iodine levels below 50 μg/L (although this level suggests moderate deficiency, it is impossible to ascertain deficiency from a single urine sample). By the time of the 2000–2001 NHANES, 7.3% of pregnant women had urinary iodine values below 50 μg/L.

Where did all the iodine go? According to the Virginia-based Salt Institute, only 20% of food salt is iodized today, and most of that is sold at grocery stores. Salt iodization is voluntary in the United States, and Morton Satin, president of the Salt Institute, says most food processors and restaurants never adopted iodized salt because it wasn’t required. As Americans have embraced processed foods and restaurant meals, non-iodized salt has supplanted the iodized type in many people’s diets. Satin says his organization has publicly asked the food and restaurant industries for broader iodization. Iodine was also used at one time in dairy products and bread, but has been replaced with more effective alternatives.

Purnendu Dasgupta, a biochemist at The University of Texas at Arlington, published research in the 15 February 2008 issue of *Environmental Science & Technology* showing that 53% of iodized salt samples tested had lower iodine levels than recommended by the U.S. Food and Drug Administration. The study also revealed that exposure to high humidity diminishes iodine in salt.

The possibility of asymptomatic iodine deficiency during pregnancy is raising concern. “I think it’s quite likely we’ve had subtle neurological deficiencies in babies born in the U.S. [to mothers with mild iodine deficiencies],” says Elizabeth Pearce, an assistant professor of medicine at Boston University School of Medicine. To protect developing fetuses, Pearce and other scientists are calling on the government to boost Americans’ iodine intake. “I wouldn’t endorse greater salt intake,” says thyroid specialist Robert Utiger of Harvard Medical School, referring to salt’s association with hypertension, heart disease, and stroke. “But the iodine content of salt ought to be increased.”

Since 1994, the World Health Organization has recommended universal salt iodization to combat deficiency. Says Satin, “One approach would be to adopt the strategy being employed in New Zealand. They are taking measured steps in specific food categories and can keep adding [iodization to] additional food categories as monitoring dictates.” Kevin Sullivan, an associate professor of epidemiology at Emory University in Atlanta, says that if food processors adopt iodized salt we should closely monitor iodine levels through ongoing NHANES studies to see if iodization recommendations need to be adjusted.

Another approach is to require iodine in all prenatal vitamins. Although the American Thyroid Association has promoted such a measure since 2006, Pearce says only an estimated 50% of prenatal vitamins contain iodine.

Says Dasgupta: “Some believe that iodine deficiency is a disease of the past that was ‘cured’ in the 1930s with the iodization practices. This leads to the thought today that iodization is not needed because it was cured, but the fact is that it is a deficiency disorder, not a disease.”

